# Complexity of Human Antibody Response to Dengue Virus: Implication for Vaccine Development

**DOI:** 10.3389/fmicb.2017.01372

**Published:** 2017-07-20

**Authors:** Wen-Yang Tsai, Hong-En Lin, Wei-Kung Wang

**Affiliations:** Department of Tropical Medicine, Medical Microbiology and Pharmacology, John A. Burns School of Medicine, University of Hawaii at Manoa Honolulu, HI, United States

**Keywords:** dengue virus, antibody, envelope protein, precursor membrane protein, epitopes

## Abstract

The four serotypes of dengue virus (DENV) are the leading cause of arboviral diseases in humans. Decades of efforts have made remarkable progress in dengue vaccine development. Despite the first dengue vaccine (dengvaxia from Sanofi Pasteur), a live-attenuated tetravalent chimeric yellow fever-dengue vaccine, has been licensed by several countries since 2016, its overall moderate efficacy (56.5–60.8%) in the presence of neutralizing antibodies during the Phase 2b and 3 trials, lower efficacy among dengue naïve compared with dengue experienced individuals, and increased risk of hospitalization among young children during the follow-up highlight the need for a better understanding of humoral responses after natural DENV infection. Recent studies of more than 300 human monoclonal antibodies (mAbs) against DENV have led to the discovery of several novel epitopes on the envelope protein recognized by potent neutralizing mAbs. This information together with in-depth studies on polyclonal sera and B-cells following natural DENV infection has tremendous implications for better immunogen design for a safe and effective dengue vaccine. This review outlines the progress in our understanding of mouse mAbs, human mAbs, and polyclonal sera against DENV envelope and precursor membrane proteins, two surface proteins involved in vaccine development, following natural infection; analyses of these discoveries have provided valuable insight into new strategies involving molecular technology to induce more potent neutralizing antibodies and less enhancing antibodies for next-generation dengue vaccine development.

## Introduction

The four serotypes of dengue virus (DENV) cause the most common and important arboviral diseases in humans (Guzman and Harris, 2015). Approximately 390 million DENV infections occur annually with 20–25% apparent infection, including dengue fever (DF), and a severe and potentially life-threatening disease, dengue hemorrhagic fever (DHF), and dengue shock syndrome (DSS) (World Health Organization, 2009; Bhatt et al., 2013; Guzman and Harris, 2015). Decades of efforts have made tremendous progress in dengue vaccine development; several candidate vaccines are currently in different phases of clinical trials (Murphy and Whitehead, 2011; Schwartz et al., 2015). Despite the Sanofi Pasteur's dengvaxia, a live-attenuated chimeric yellow fever-dengue tetravalent dengue vaccine (CYD-TDV), has been licensed as the first dengue vaccine by several countries since 2016, the moderate efficacy (56.5–60.8%) in the presence of neutralizing antibodies (Abs) during its Phase 2b and 3 trials, lower efficacy among dengue naïve compared with dengue experienced individuals (35.5–43.2 vs. 74.3–83.7%), and increased risk of hospitalization and severe dengue among young children during the follow-up in years 3–6 highlight the need for a better understanding of immunity, in particular humoral responses, after natural DENV infection (Sabchareon et al., 2012; Capeding et al., 2014; Guy et al., 2015; Hadinegoro et al., 2015; Thomas, 2015; Villar et al., 2015; Aguiar et al., 2016; Flasche et al., 2016; Halstead, 2016; Halstead and Russell, 2016; Wilder-Smith et al., 2016). Recent studies of more than 300 human monoclonal antibodies (mAbs) have led to the discovery of several potent neutralizing epitopes; these together with detailed analysis of polyclonal sera and B-cells after natural infection have provided valuable insights into dengue vaccine development. This review outlines the progress in our understanding of the epitopes and specificity of mouse mAbs, human mAbs and polyclonal sera against DENV surface proteins, the envelope (E) and precursor membrane (prM) proteins, following natural infection; and their implication for new strategies of dengue vaccine to induce more potent neutralizing Abs and less enhancing Abs.

## Genome, virion structure, assembly, and maturation

The genome of DENV consists of a positive-sense, single-stranded RNA of 10.6 kb in length. Flanked by the 5′ and 3′ untranslated regions, the single open reading frame encodes a polyprotein precursor, which is cleaved by cellular and viral protease into three structural proteins, the capsid (C), prM, and E, and seven non-structural (NS) proteins, NS1, NS2A, NS2B, NS3, NS4A, NS4B, and NS5 (Murphy and Whitehead, 2011; Pierson and Diamond, 2013).

The E protein, ~55 kDa in size, contains two N-linked glycans and 12 strictly conserved cysteines forming 6 disulfide bridges (Pierson and Diamond, 2013). It contains 495 (DENV1, 2, 4) or 493 (DENV3) amino acids. As the major surface protein present on virions, E protein participates in virus entry and is the main target of neutralizing and enhancing Abs (Murphy and Whitehead, 2011; Pierson and Diamond, 2013). The ectodomain at the N-terminus contains three distinct domains. Domain I (DI) is located in the center, domain II (DII), an elongated domain containing the fusion loop at its tip, is involved in dimerization and membrane fusion, and domain III (DIII), an immunoglobulin-like domain, is involved in receptor binding and stabilization of trimers during fusion (Modis et al., 2004; Pierson and Diamond, 2013). At the C-terminus, there are two α-helices in the stem region and two transmembrane domains in the anchor region (Zhang W. et al., 2003; Pierson and Diamond, 2013).

The prM protein, ~19 kDa in size, contains one N-linked glycan and 6 highly conserved cysteines forming 3 disulfide bridges (Pierson and Diamond, 2013). The prM protein consists of 166 amino acids; during maturation of virions it is cleaved into the pr peptide (91 residues) and membrane (M) protein (75 residues), which includes an N-terminal loop, an α-helical domain, and two transmembrane domains (Pierson and Diamond, 2013; Zhang et al., 2013). After biosynthesis, prM protein forms a heterodimer with E protein to function as a chaperone for E protein and prevent premature fusion of E protein within acidic compartments along the secretory pathway (Mukhopadhyay et al., 2005; Pierson and Diamond, 2013).

**Figure 1:**
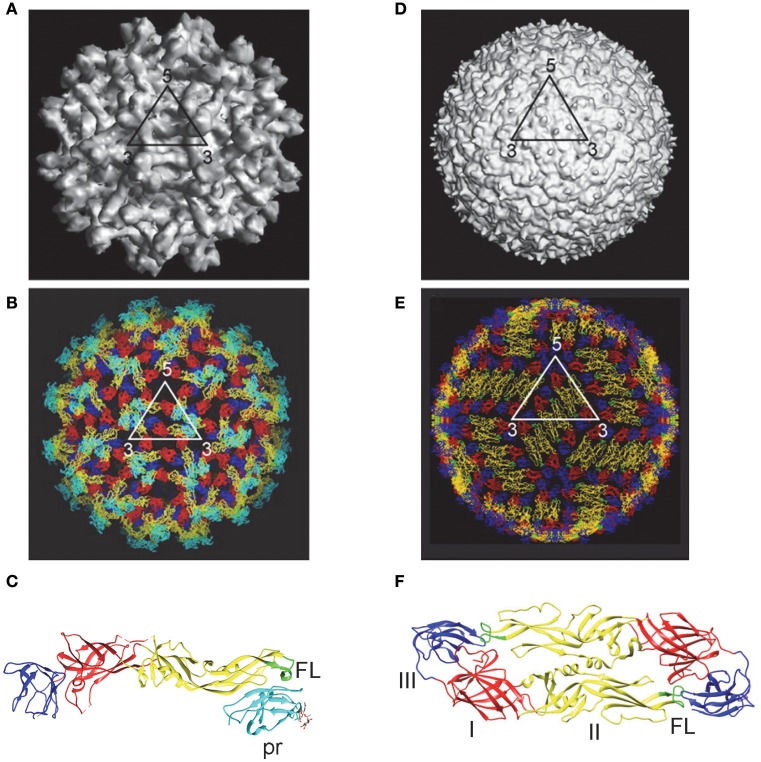
Immature and mature DENV particles. Immature particles: **(A)** Cryo-EM picture showed spiky surface, formed by 60 trimeric prM-E heterodimers. **(B)** Pseudoatomic structure showed pr peptide (cyanine) and fusion loop (FL, green) on surface (Zhang Y. et al., 2003; Perera et al., 2008). **(C)** X-ray crystal structure of prM-E heterodimers showed pr peptide (cyanine) and FL (green) exposed on each spike (Li et al., 2008). Mature particles: **(D)** Cryo-EM picture showed smooth surface with 90 E dimers in herringbone pattern. **(E)** Pseudoatomic structure showed poorly exposed FL (green) (Kuhn et al., 2002; Perera et al., 2008). **(F)** X-ray crystal structure of E protein ectodomain (E-E dimers) revealed DI (red), DII (yellow) with its FL (green), and DIII (blue) (Modis et al., 2004). (**A,B,D,E** with permission from the authors of Perera et al., 2008; **C,F** generated by the program UCSF Chimera).

DENV enters the cell through receptor mediated endocytosis. The low-pH environment in endosome results in conformational change of E protein and fusion between viral and endosomal membranes (Modis et al., 2004; Mukhopadhyay et al., 2005; Kielian and Rey, 2006; Pierson and Diamond, 2013). The assembly of DENV occurs in the membranes derived from rough ER, where the immature virions bud into the lumen of ER and transport through the secretary pathway (Mukhopadhyay et al., 2005; Pierson and Diamond, 2013). Within the low pH environment of trans-Golgi the prM protein on immature virions is cleaved by furin or furin-like protease into pr peptide and M protein; pr is released under neutral pH in the culture medium to form mature particles (Perera and Kuhn, 2008; Yu et al., 2008). However, the prM cleavage is inefficient, leading to a mixture of mature, immature and partially immature DENV particles in tissue culture (Cherrier et al., 2009; Junjhon et al., 2010). Cryo-EM studies have revealed structural details of mature and immature DENV particles as shown in Figure 1.

## DENV proteins recognized by Abs after DENV infection

Following primary DENV infection, individuals develop an IgM response, followed by an IgG response. After secondary DENV infection, individuals develop a greater IgG response compared to IgM response (Innis, 1997; Vaughn et al., 1999). IgM response is relevant to dengue diagnosis, whereas IgG response is critical to pathogenesis, protection or enhancement, and vaccine development.

**Figure 2:**
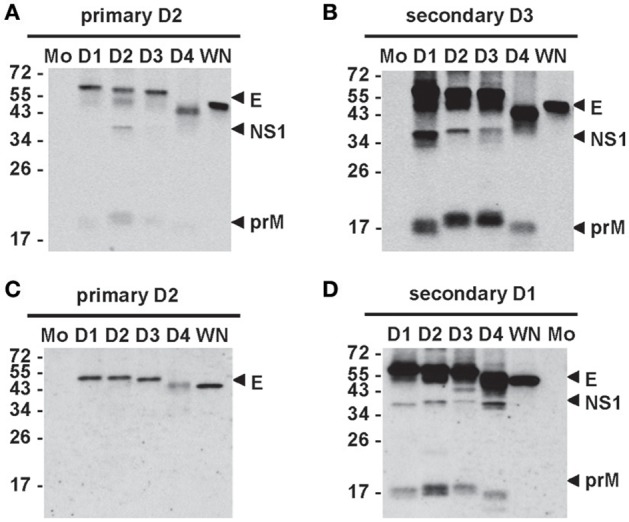
Antibody responses after primary and secondary DENV infections. Sera from cases with primary **(A)** and secondary **(B)** DENV infections and sera from vaccinees receiving primary immunization **(C)** with a live-attenuated DENV2 vaccine and secondary immunization **(D)** with another live-attenuated DENV1 vaccine were subjected to WB analysis using virus-infected cell lysates (Tsai et al., 2015). Mo, mock; D1, DENV1; D2, DENV2; D3, DENV3; D4, DENV4; WN, WNV.

Different assays have been employed to study Abs response after DENV infection, including enzyme-linked immunosorbent assay (ELISA), Western blot (WB) and microsphere immunoassay to examine binding Abs, as well as neutralization and enhancing assays for functional Abs. Most binding assays, which use either DENV virions or recombinant E protein as antigen, provide little information on Abs targeting different DENV proteins, whereas WB analysis that employs antigens derived from DENV-infected cells can detect Abs responses to individual DENV proteins and their relative abundance. Early studies reported E, NS3, and NS5 proteins were recognized (Churdboonchart et al., 1991; Se-Thoe et al., 1999; Valdes et al., 2000). Later studies revealed that the strongest Abs response was anti-E Abs which recognize both the infecting DENV serotype and three other serotypes plus West Nile virus (WNV), a member of Japanese encephalitis virus (JEV) serocomplex, followed by anti-prM and anti-NS1 Abs after primary DENV infection (Figure 2A). Following secondary infection, strong cross-reactive anti-E Abs plus anti-prM and anti-NS1 Abs were found (Figure 2B) (Lai et al., 2008; Tsai et al., 2015). A similar trend was observed in vaccinees following primary and secondary immunization with monovalent live-attenuated DENV vaccines (Figures 2C,D). Together, these observations suggest that cross-reactive anti-E Abs are the major Abs, followed by anti-prM and NS1 Abs.

## Different categories of anti-E mAbs: binding specificity and epitopes

In the genus *Flavivirus* of the family *Flaviviridae*, members belonging to different serocomplexes cause significant human diseases, including the four serotypes of DENV in the DENV serocomplex, WNV and JEV in the JEV serocomplex, tick-borne encephalitis virus (TBEV) in the TBEV serocomplex, YFV as a single member, and Zika virus (ZIKV) in its serocomplex (Pierson and Diamond, 2013). The amino acid sequence homology of E protein is about 39–49% between different serocomplexes, 63–78% between DENV serotypes, and up to 96–97% between genotypes within each DENV serotype (Stiasny et al., 2006). Based on the binding specificity, anti-E Abs that recognize members of two or more serocomplexes, members within the same serocomplex, or a single member are categorized as group-reactive, complex-reactive, and type-specific Abs, respectively (Calisher et al., 1989). Determining the binding specificity of anti-DENV Abs provides important information on the extent of cross-reactivity to different flaviviruses, but this requires the inclusion of 4 DENV serotypes and other flaviviruses as antigens in the binding assay. Most studies utilized antigens from 4 serotypes only and classified anti-DENV Abs as type-specific and cross-reactive Abs, which include both complex-reactive and group-reactive Abs.

DENV E protein contains both linear and discontinuous epitopes. Linear epitopes can be identified by peptide-scan ELISA; discontinuous epitopes can be identified by alanine-scanning or shortgun mutagenesis, yeast or phage-display library, neutralization escape mutant, and cryo-EM analysis of Fab and virions. Although a few linear epitopes on E protein have been reported based on peptide ELISA of dengue-immune sera (Aaskov et al., 1989; Megret et al., 1992; da Silva et al., 2009), the majority of anti-E Abs likely recognize discontinuous and conformational epitopes. The evidence came from studies of mouse anti-E mAbs and anti-E Abs in human sera, in which most lost binding in WB analysis under reducing condition and were thus sensitive to the conformation provided by disulfide bridges (Megret et al., 1992; Roehrig et al., 1998; Lai et al., 2008). Consistent with this, a mutational study revealed that the 6 disulfide bridges formed by 12 cysteines were critical for the epitope expression and conformation of DENV E protein (Roehrig et al., 2004).

## Epitopes recognized by mouse anti-E mAbs

Studies of mouse mAbs against DENV have shown that different categories of anti-E mAbs have different epitopes and neutralizing potency. Group-reactive mAbs primarily recognize the highly conserved residues in the fusion loop of DII, whereas complex-reactive and type-specific mAbs recognize different but slightly overlapping residues in DIII (Crill and Chang, 2004; Gromowski and Barrett, 2007; Gromowski et al., 2008; Lin et al., 2012). The neutralizing potency of type-specific mAbs was generally higher than that of complex-reactive mAbs, which in turn was higher than that of group-reactive mAbs (Gromowski and Barrett, 2007; Sukupolvi-Petty et al., 2007; Gromowski et al., 2008). Several type-specific anti-DIII potent neutralizing mAbs (NT_50_ in the range of ng/ml) have been reported as potential therapeutic mAbs (Brien et al., 2010; Shrestha et al., 2010; Sukupolvi-Petty et al., 2010, 2013). It should be noted that these potent neutralizing mAbs were generated by an immunization protocol including two DENV infections in the IFN-α/β receptor-deficient (IFN-αβR^−/−^) strain in the C57BL/6 background followed by boost with recombinant DIII (rDIII), which was different from early protocols using DENV to immunize wild type (WT) BALB/c mice (Gromowski and Barrett, 2007; Sukupolvi-Petty et al., 2007; Lin et al., 2012). More than 200 mouse anti-E mAbs including several potent neutralizing mAbs are summarized in Table 1.

**Table 1:** Summary of mouse anti-E mAbs reported in four large studies.

**Immunogen^a^**	**# of mAbs**	**Specificity^b^ TS/CrR[CR+GR]**	**Binding^c^ DI/II DIII E**		**Epitopes^d^**	**Potent NT mAbs or remarks^e^**	**References**
DENV1	67	30 TS (45%) 30 CrR (45%) 7 ND	5 5 4	25 25 3	DIII: lr, str A and str G	15 strong NT mAbs 14 anti-DIII	Shrestha et al., 2010
DENV2	33	20 TS (61%) 11 CrR (33%) 2 ND	8 6 2	11 1 5	DIII: lr, CCL and str A, DI: lr, DII: lr, di and FL	24 strong NT mAbs 11 anti-DIII, 13 anti-DI/DII	Sukupolvi-Petty et al., 2010
DENV3	74	48 TS (65%) 24 CrR (32%) 2 ND	13 15 2	25 10 4 5	DIII: lr, str A and str G	22 strong NT mAbs 19 anti-DIII	Brien et al., 2010
DENV4	47	26 TS (55%) 17 CrR (36%) 4 ND	6 9 2	13 7 5 3 2	DIII: lr, CCL, str F and str G	6 strong NT mAbs 5 anti-DIII	Sukupolvi-Petty et al., 2013

a *Immunization protocol: IFN-αβR^−/−^ C57BL/6 mice infected with DENV twice, and boosted with rDIII once*.

The method of generating mouse mAbs was the same, including isolation of splenocytes, hybridoma and

*screening by flow cytometry with DENV-infected cells*.

b *TS: type-specific, CrR: cross-reactive, CR: complex-reactive, GR: group-reactive*.

c *Binding to recombinant DI/II or DIII of E protein or E protein, ND: not done*.

d *lr: lateral ridge, str: strand, CCL: CC' loop, di: dimer interface, FL: fusion loop*.

e *NT: neutralization. Strong NT mAbs were defined by NT >90% at 10 μg/ml (Brien et al., 2010; Shrestha et al., 2010; Sukupolvi-Petty et al., 2010, 2013). Individual potent NT mAbs (NT_50_ < 0.5 μg/ml) are not listed*.

Several in-depth studies of mouse anti-E mAbs against DENV and WNV have provided critical insights into the mechanisms of neutralization including the stoichiometry (Pierson et al., 2007), maturation status (Nelson et al., 2008), temperature (Dowd et al., 2011), step of fusion (Thompson et al., 2009), genotype/strain difference (Austin et al., 2012; Sukupolvi-Petty et al., 2013), type-specific or cross-neutralization (Cockburn et al., 2012a,b; Midgley et al., 2012), structural organization, and “breathing” on virion (Lok et al., 2008; Edeling et al., 2014).

## Epitopes recognized by human anti-E mAbs

Several technologies including EBV-immortalization of B cells, cytofusion-hybridoma, and single cell-expression cloning of plasmablasts (Wilson and Andrews, 2012) have been employed to generate human mAbs against DENV after natural infection or vaccination (Beltramello et al., 2010; de Alwis et al., 2011, 2012; Smith et al., 2012, 2013a,b, 2014; Teoh et al., 2012; Xu et al., 2012; Costin et al., 2013; Tsai et al., 2013; Dejnirattisai et al., 2015; Priyamvada et al., 2016). Table 2 summarizes more than 300 human anti-E mAbs reported thus far. Despite different methods/antigens were used to generate and characterize these mAbs, predominant cross-reactive mAbs were identified from individuals after primary and secondary DENV infection, whereas type-specific mAbs were identified mainly from individuals after primary infection.

**Table 2:** Summary of human anti-E mAbs reported in literature.

**Methods^a^**	**Immune status of hosts**	**# of mAbs**	**Specificity^b^ TS/CrR[CR+GR]**	**Binding^c^ DI/II DIII**		**Epitopes^d^**	**Potent NT mAbs or remarks^e^**	**References**
Memory BC EBV-imm, V-cell flow, V-cell ELISA	3 primary infections 2 secondary infections	20 27	12 TS (60%) 8 CrR (40%) 1 TS (4%) 26 CrR (96%)	8 4 1 22	4 4 0 4		several TS anti-DIII and CrR anti-DI/DII	Beltramello et al., 2010
Memory BC EBV-imm, V-cell flow	2 primary infections	11	5 TS (45%) 6 CrR (55%)	ND ND	4 2	303, 304, 305, 307, 310, 317, 384	3.7, 25.5,10.16,35.3 18.21,13.6,23.13	de Alwis et al., 2011
Memory BC EBV-imm, V-ELISA	1 primary infection	1	1 TS	ND	ND	quaternary epitope	HM14c10	Teoh et al., 2012
Memory BC hybridoma V-ELISA	5 primary infections 5 secondary infections	25 5	2 TS (8%) 23 CrR (92%) 0 TS (0%) 5 CrR (100%)	1 19 4	1 4 1	quaternary epitope	2D22, 5J7	Smith et al., 2012
Memory BC Hybridoma, V-ELISA	14 primary immunizations 4 primary infections	16 24	0 TS (0%) 16 CrR (100%) 1 TS (4%) 23 CrR (96%)	9 0 16	7 1 7			Smith et al., 2013b
Memory BC, EBV-imm, or PCR-EC, V-ELISA	1 secondary infection 2 primary infections	3	3 CrR	3	0	101, 109	1.6D	Costin et al., 2013
Memory BC EBV-imm, V-cell flow, plasmablasts, SC-EC, V-ELISA	4 primary Infections 4 secondary Infections	28 23	10 TS (36%) 18 CrR (64%) 0 TS (0%) 23 CrR (100%)	ND ND ND ND	ND ND ND ND	GR mAbs: 101, 106, 107, 108, 76, 78 GR mAbs:101, 106, 107, 108, 76, 78	GR mAbs: FL or FL+bc loop	Tsai et al., 2013
Memory BC Hybridoma, V-ELISA	6 primary infections 5 secondary infections	9 21	9 CrR 21 CrR	ND ND	ND ND	101, 106, 107, 108, 110, 111, 104 73, 78, 79	1M7 1C19: bc loop 1N5	Smith et al., 2013a
Memory BC Hybridoma, V-ELISA	2 primary infections 1 secondary infection	11 23	3 TS (27%) 8 CrR (73%) 2 TS (9%) 21 CrR (91%)	0 2 0 20	2 2 1 0	I/II hinge	3F9, 1L12 1M7 1F4	Smith et al., 2014
Plasmablasts SC-EC, V-ELISA	1 primary infection 6 secondary infections	32 113	2 TS (6%) 30 CrR (94%) 2 TS (2%) 111 CrR (98%)	ND ND ND ND	ND ND ND ND	EDE2 EDE1, EDE2 or FLE EDE2 EDE1, EDE2 or FLE	50 EDE mAbs more potent NT than 46 FL mAbs	Dejnirattisai et al., 2015

a *BC, B-cells; EBV-imm, Epstein-Barr virus immortalization; V-cell flow, screen by flow cytometry using virus-infected cells; V-cell ELISA, screen by ELISA using virus-infected cells; V-ELISA, screen by ELISA using virion, PCR-EC; PCR expression cloning of Ig genes; SC-EC, Single-cell PCR expression cloning of Ig genes*.

b *TS, type-specific; CrR, cross-reactive; CR, complex-reactive; GR, group-reactive*.

c *Binding to recombinant DI/II or DIII of E protein, ND: not done*.

d *Major epitopes identified are listed. EDE, E dimer epitope; FLE, fusion loop epitope (Dejnirattisai et al., 2015)*.

e *NT, neutralization; FL, fusion loop. Potent NT mAbs with NT_50_ < 0.5 μg/ml are listed*.

**Figure 3:**
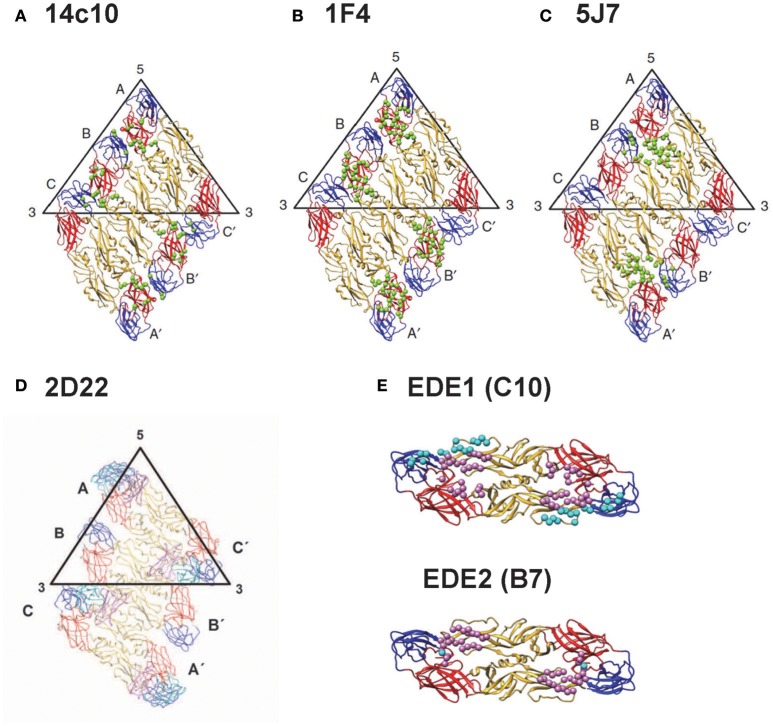
Epitopes recognized by potent human anti-E mAbs. **(A)** DENV1- type specific (TS) mAb (14c10) recognizes quaternary epitopes involving DI, DII, and DIII on virion (Teoh et al., 2012). **(B)** Another DENV1-TS mAb (1F4) recognizes monomeric DI/II hinge present on virion (de Alwis et al., 2012; Fibriansah et al., 2014; Smith et al., 2014). **(C)** DENV3-TS mAb (5F7) recognizes quaternary epitopes involving DI, DII, and DIII on virion (de Alwis et al., 2012; Smith et al., 2012; Fibriansah et al., 2015b). **(D)** DENV2-TS mAb (2D22) recognizes quaternary epitopes involving DI, DII and DIII on virion (de Alwis et al., 2012; Smith et al., 2012; Fibriansah et al., 2015a). **(E)** cross-reactive mAbs C10 and B7 recognize E-dimer epitope 1 (EDE1) and E-dimer epitope 2 (EDE2), respectively; both involve fusion loop and other residues on virion (Dejnirattisai et al., 2015; Fibriansah et al., 2015a; Rouvinski et al., 2015). (Panels **A–E** with permission from the authors of Fibriansah et al., 2015a,b).

Based on the binding to recombinant E protein, DIII or DI/II, alanine mutants, yeast library or escape mutants, group-reactive mAbs were found to recognize either residues in fusion loop or both fusion loop and bc loop, a loop next to fusion loop in DII (Costin et al., 2013; Smith et al., 2013a; Tsai et al., 2013), type-specific mAbs recognize epitopes in DIII, interdomain residues, DI/II hinge region or quaternary epitopes on virion (Beltramello et al., 2010; de Alwis et al., 2011, 2012; Teoh et al., 2012; Fibriansah et al., 2014, 2015a,b), and complex-reactive mAbs recognize DIII or E-dimer epitope which involves fusion loop and other residues (Beltramello et al., 2010; de Alwis et al., 2011; Dejnirattisai et al., 2015). The quaternary epitopes recognized by several type-specific potent neutralizing mAbs (14C10, 1F4, 2D22, and 5F7) are shown in Figures 3A–D (Teoh et al., 2012; Fibriansah et al., 2014, 2015a,b); the E-dimer epitopes 1 and 2 recognized by complex-reactive potent neutralizing mAbs are shown in Figure 3E (Rouvinski et al., 2015).

## Epitopes recognized by anti-prM mAbs

After the early reports of some mouse anti-prM mAbs (Megret et al., 1992; Roehrig et al., 1998), a large panel of human anti-prM mAs were found to be cross-reactive, weakly or non-neutralizing, and enhance DENV in FcγR cells, suggesting anti-prM response is detrimental (Dejnirattisai et al., 2010). Consistent with this, mouse anti-prM mAbs were reported to cause antibody-dependent enhancement (ADE) *in vitro*, especially for immature or partially immature DENV (Rodenhuis-Zybert et al., 2010). Since fusion loop epitopes were more exposed on immature DENV, anti-fusion loop mAbs were shown to preferentially bind immature DENV (Cherrier et al., 2009), enhance their infectivity *in vitro* (Rodenhuis-Zybert et al., 2011), and cause ADE *in vivo*, including monkey (Goncalvez et al., 2007) and lethal disease in AG129 mice (Shresta et al., 2006; Balsitis et al., 2010; Zellweger et al., 2010).

The epitopes of mouse anti-prM mAbs were previously reported in a conserved region of pr peptide (residues 53–67) (Huang et al., 2006). A recent study of 25 human anti-prM mAbs revealed several overlapping epitopes within a major antigenic site in the pr peptide; a few anti-prM mAbs can recognize E protein as well (Chan et al., 2012; Smith et al., 2015).

## Different categories of anti-E Abs in polyclonal human serum

Previously, depletion of human sera with antigen derived from non-infecting DENV serotypes, so called heterologous serotypes, reported that the majority of anti-E Abs after primary infection was cross-reactive and a minor proportion was type-specific (Lai et al., 2008). This was supported by studies using WT and mutant virus-like particles (VLPs) containing mutations of cross-reactive epitopes to measure the endpoint ELISA titers and determine the proportion of cross-reactive and type-specific Abs (Crill et al., 2009; Lai et al., 2013). Another study using rDIII in depletion revealed the proportion of type-specific anti-DIII Abs was 0.4–8.1% after primary DENV infection (Wahala et al., 2009). Interestingly, an integrated analysis of 319 human anti-E mAbs from 7 studies in Table 2, of which each contained a panel of (rather than one or two) human mAbs to represent the Ab repertoire in the study subjects, revealed that the percentages of cross-reactive vs. type-specific mAbs derived from primary and secondary DENV infections were 76.8 vs. 23.2% and 97 vs. 3%, respectively (Table 3). Consistent with this, a recent study using multicolor fluorospot assay to examine memory B cell population reported that the percentages of cross-reactive vs. type-specific B-cell clones after primary and secondary infections were 52 vs. 48% and 98 vs. 0.2–2%, respectively (Hadjilaou et al., 2015).

**Table 3:** Relationship between the specificity of human anti-E mAbs and immune status of hosts.

**Immune status of hosts**	**# of mAbs**	**Specificity of mAbs**				**References^b^**
		**TS^a^**	**CrR^a^ [CR+GR]**	**CR^a^**	**GR^a^**	
Primary DENV infection (*n* = 21)	151	35 (23.2%)	116 (76.8%)	57 (37.7%)	59 (39.1%)	Beltramello et al., 2010; de Alwis et al., 2011; Smith et al., 2012, 2013a, 2014; Tsai et al., 2013; Dejnirattisai et al., 2015
Secondary DENV infection (*n* = 14)	168	5 (3.0%)	163 (97.0%)	71 (42.3%)	92 (54.7%)	Beltramello et al., 2010; Smith et al., 2012, 2014; Tsai et al., 2013; Dejnirattisai et al., 2015
Total (*n* = 35)	319	40 (12.5%)	279 (87.5%)	128 (40.1%)	151 (47.3%)	Beltramello et al., 2010; de Alwis et al., 2011; Smith et al., 2012, 2013a, 2014; Tsai et al., 2013; Dejnirattisai et al., 2015

a *TS, type-specific; CrR, cross-reactive; CR, complex-reactive; GR, group-reactive. For studies that did not separate CrR mAbs into CR and GR mAbs, the CrR mAbs that bind DI/II are considered GR mAbs and those bind DIII are considered CR mAbs in this analysis*.

b *Studies in Table 2 that characterized the binding specificity of a panel of human mAbs derived from individuals with known DENV immune status were included in the analysis*.

## Epitopes recognized by neutralizing anti-E Abs in polyclonal serum

It is known that following primary DENV infection, individuals develop monotypic neutralizing Abs against the infecting serotype, which correlate with the long-lived protection against that serotype (Sabin, 1952; Innis, 1997; Halstead, 2003; Imrie et al., 2007). After recovery from secondary infection, individuals develop multitypic neutralizing Abs not only against the previously exposed serotypes but also against the serotypes to which they have not yet been exposed (“non-exposed” serotypes) (Innis, 1997; Halstead, 2003). Such heterotypic neutralizing Abs are thought to contribute to protection against the non-exposed serotypes during the third or fourth DENV infections, as suggested by the lower rates of hospital admission (Gibbons et al., 2007) and reduced risk of symptomatic DENV infection in humans (Olkowski et al., 2013) as well as lower viremia in monkeys (references in Tsai et al., 2013).

Several studies have investigated the nature of anti-E Abs contributing to neutralizing activities after DENV infection. In the situation of primary infection, an initial study reported that rDIII cannot deplete the monotypic type-specific neutralizing activity after primary infection, suggesting that anti-DIII Abs do not contribute to such neutralizing activity (Wahala et al., 2009). A second study showed that DENV virions of the infecting serotype rather than those of the non-exposed serotypes or recombinant E proteins can deplete the monotypic type-specific neutralizing activity, suggesting that type-specific anti-E Abs recognizing epitopes present on virions contribute to such neutralizing activity (de Alwis et al., 2012). Analysis of the escape mutants of 3 potent TS human mAbs (1F4, 2D22, and 5F7) that bind to virions revealed their epitopes on the DI/II hinge region or DIII (de Alwis et al., 2012), which was further verified by cryo-EM studies (Fibriansah et al., 2014, 2015a,b) (Figures 3B–D). A study of vaccinees reported reduced neutralization sensitivity to mutant (E126K/E157K) DENV1 replicon particles in 18 (77%) of 24 vaccinees receiving live-attenuated DENV1 vaccine, suggesting anti-E Abs in polyclonal serum recognizing these two residues proximal to the DI/II hinge contribute to the type-specific neutralizing activities (VanBlargan et al., 2013). This was followed by a series of studies using chimeric viruses, in which the candidate epitope residues on E protein were replaced by those of other serotypes. A study of chimeric virus containing 25 DI/II hinge residues of DENV4 in the DENV3 backbone (rDENV3/4) reported loss of neutralization in 4 primary DENV3 sera and gain of neutralization in 4 primary DENV4 sera (Messer et al., 2014), though the article was retracted due to contamination of rDENV3/4 virus stock (Messer et al., 2015). A second study using chimeric virus containing 40 DIII residues of DENV2 in the DENV4 backbone (rDENV4/2) revealed gain of neutralization in 7 primary DENV2 sera and no loss of neutralization in 4 primary DENV4 sera, suggesting these residues contribute to type-specific neutralizing activity after primary DENV2 but not DENV4 infection (Gallichotte et al., 2015). A recent study using chimeric virus containing 25 DI/II hinge residues of DENV3 in the DENV4 backbone (rDENV4/3) revealed >60% loss of neutralization in 3 out of 6 primary DENV4 sera and all 4 vaccinees receiving live-attenuated DENV4 vaccine, suggesting these residues contribute to type-specific neutralizing activity after primary DENV4 infection/immunization (Nivarthi et al., 2017).

Taken together, these studies suggest that type-specific Abs recognizing residues proximal to DI/II hinge, DIII complex epitope residues, and DI/II hinge residues contribute to type-specific neutralizing activity following primary DENV1 immunization, primary DENV2 infection/immunization, and primary DENV4 infection/immunization, respectively (VanBlargan et al., 2013; Gallichotte et al., 2015; Nivarthi et al., 2017). It is worth noting that type-specific neutralizing Abs in 23% of individuals after primary DENV1 immunization do not recognize residues proximal to DI/II hinge, suggesting the presence of other type-specific neutralizing epitope in some DENV1 vaccinees (VanBlargan et al., 2013). Similarly, 32% of type-specific neutralizing Abs after primary DENV2 infection/immunization recognize DIII residues alone, suggesting the presence of type-specific neutralizing epitopes other than DIII complex epitope in DENV2 immune serum (Gallichotte et al., 2015). The observation that 50% (3 out of 6) of individuals after primary DENV4 infection do not recognize the DI/II hinge residues suggests the presence of other type-specific neutralizing epitopes in DENV4 immune serum (Nivarthi et al., 2017). This is in agreement with the reports of individual variations in the specificity of anti-E Abs to different domains and their contribution to neutralizing activities in polyclonal sera after YFV vaccination and TBEV infection/immunization (Vratskikh et al., 2013; Jarmer et al., 2014).

In the situation of secondary DENV infection, it was reported that group-reactive anti-E mAbs derived from patients after secondary DENV infection had higher binding avidity and neutralizing potency compared with those derived after primary infection, suggesting cross-reactive anti-E Abs (including group-reactive and complex-reactive Abs) evolved from low avidity and poor neutralizing after primary infection to high avidity and potent neutralizing after secondary infection (Tsai et al., 2013). A recent study using a two-step depletion protocol to remove group-reactive and complex-reactive anti-E Abs revealed that cross-reactive anti-E Abs contributed significantly to neutralizing activities against both exposed and non-exposed DENV serotypes after secondary DENV infection/immunization (Tsai et al., 2015).

## Epitopes recognized by enhancing Abs in polyclonal serum

Previous epidemiological studies have shown that individuals experiencing a secondary DENV infection had a significant higher risk of severe disease than those experiencing primary infection (Halstead, 1988 and references in). In the presence of diluted dengue immune serum, DENV replicates to produce higher titers of virus particles in human Fcγ receptor-bearing cells compared with that in the absence of serum, a phenomenon known as ADE (Halstead and O'Rourke, 1977; Halstead, 1988, 2003). Further studies have shown that ADE is a concentration-dependent phenomenon and can be caused by both non-neutralizing mAbs and neutralizing mAbs at suboptimal concentration (Pierson et al., 2007, 2008; Nelson et al., 2008), including very potent human neutralizing mAbs (Beltramello et al., 2010; Teoh et al., 2012; Fibriansah et al., 2014, 2015a,b; Dejnirattisai et al., 2015). Thus, induction of potent and durable neutralizing Abs has been a goal of DENV vaccine development (Murphy and Whitehead, 2011; Schwartz et al., 2015).

Using depletion protocol together with K562 cell-based ADE assay and mouse model, a recent study demonstrated that removal of cross-reactive Abs in primary DENV-immune sera ablated ADE *in vitro* and *in vivo* (de Alwis et al., 2014). Moreover, both recombinant E-specific Abs (including fusion loop Abs) and anti-prM Abs were shown to contribute significantly to ADE *in vitro*.

## T-cell responses after natural DENV infection

T-cell responses after DENV infection and their role in protection or pathogenesis have been reviewed previously (Rothman, 2011; Weiskopf and Sette, 2014). Early studies of T-cell responses during acute DENV infection in patients with different disease severity suggested cross-reactive CD8 T-cells contribute to pathogenesis rather than protection (Mongkolsapaya et al., 2003; Duangchinda et al., 2010). However, other studies reported that the timing of T-cell response does not match with plasma leakage, a key feature of DHF/DSS (Dung et al., 2010). Recent studies suggested protective roles of CD8 T-cells response and the association of multifunctional CD8 T-cells with protection against disease in a Sri Lankan population (Weiskopf et al., 2013; references in Rothman, 2011). To further address the role of T-cells in protection or pathogenesis, investigating the pre-existing T-cell response and disease outcome in well-designed prospective cohorts is needed (Rothman, 2011).

Since CD8 T-cell epitopes were mapped primarily to NS proteins such as NS3 and NS5 (Mathew et al., 1996; Weiskopf et al., 2013, 2015), the moderate efficacy of CYD-TDV vaccine has been attributed to the lack of NS proteins of DENV to elicit CD8 T-cell responses (Thomas, 2015; Halstead and Russell, 2016). It is worth noting that structural proteins (C, prM and E) do contain CD8 T-cell epitopes (Mathew et al., 1996; Weiskopf et al., 2013, 2015) and T-cell response to DENV structural proteins has been shown in naïve individuals receiving CYD-TDV vaccine (Harenberg et al., 2013). Subunit candidate vaccine containing recombinant DENV E protein can induce T-cell response based on IFNγ ELISPOT assays (Manoff et al., 2015). Moreover, two successful flavivirus vaccines (JEV and TBEV vaccines) are based on inactivated virions in the absence of NS proteins (Ishikawa et al., 2014). Although it remains to be investigated whether NS proteins are required to be a component of dengue vaccine, evaluation of dengue vaccine in clinical trials should include the assessment of T-cell responses (Rothman et al., 2015).

## Implication for vaccine strategy

Several candidate dengue vaccines are currently in different phases of clinical trials, including various formats such as live attenuated virus, purified inactivated virus, recombinant E protein, and DNA vaccine (Guzman and Harris, 2015; Schwartz et al., 2015). The goals are to provide long-lasting protection against each of the four serotypes, good safety profile, well-tolerated reactogenicity, suitable regimen for target population and affordable cost (Murphy and Whitehead, 2011). Following the reports of the Phase 2b and 3 trials of CYD-TDV vaccine and its subsequent licensure, several comments and reviews have been published regarding the population benefits and potential risks of this vaccine (Thomas, 2015; Halstead, 2016; Halstead and Russell, 2016; Wilder-Smith et al., 2016), implication to dengue immunopathogenesis (Screaton et al., 2015), complexity of vaccine and assays for evaluation (Flipse and Smit, 2015), and comparison with another promising candidate vaccine (Whitehead, 2016).

Both Abs and T-cell response contribute to protection and clearance of DENV infection. From the observations of natural DENV infection, reduction in DHF/DSS during early infancy less than 6 months, and passive Abs transfer experiments in animals, neutralizing Abs likely play a major role in protection (review by Murphy and Whitehead, 2011). However, non-neutralizing Abs and neutralizing Abs at suboptimal concentrations can cause ADE *in vitro* and *in vivo* (Goncalvez et al., 2007; Pierson et al., 2007, 2008; Nelson et al., 2008; Balsitis et al., 2010; Zellweger et al., 2010; Murphy and Whitehead, 2011). Thus, induction of durable potent neutralizing Abs and less enhancing Abs has been a goal of DENV vaccine development (Murphy and Whitehead, 2011; Schwartz et al., 2015). In this regard, the discovery of epitopes recognized by potent neutralizing mAbs following natural DENV infection have important implication for dengue vaccine development. These potent neutralizing epitopes include DIII, DI/II hinge region, quaternary epitopes on virion, E-dimer epitope, and fusion loop epitope recognized by human mAbs after secondary infection. The identification of epitopes recognized by mAbs that are weakly or non-neutralizing and enhancing, such as anti-prM mAbs, have implication for new strategy of dengue vaccine as well.

Previous reports of potent neutralizing anti-DIII mAbs in mice suggest DIII can be a potential vaccine candidate (Brien et al., 2010; Shrestha et al., 2010; Sukupolvi-Petty et al., 2010, 2013). While anti-DIII Abs represent only a small percentage of total anti-E Abs and do not contribute greatly to neutralizing activities in human sera (Crill et al., 2009; Wahala et al., 2009; Gallichotte et al., 2015), these observations do not preclude rDIII from being a vaccine candidate. This is because a number of potent neutralizing anti-DIII mAbs were also found in humans (Beltramello et al., 2010; de Alwis et al., 2011), suggesting such neutralizing Abs can be induced during natural DENV infection. Notably, an elegant study of WNV vaccine in mice showed that rDIII induced much lower neutralizing Abs compared with inactivated virions, whereas inactivated virions boosted with rDIII induced highest neutralizing Abs (Zlatkovic et al., 2011). How to induce high titers of neutralizing Abs by rDIII remains to be explored for the development of a subunit DENV vaccine.

The discovery of quaternary epitopes that are recognized by type-specific human potent neutralizing mAbs (14c10, 1F4, 2D22, and 5J7) suggests the importance of conformation and arrangement of E protein on virions (or VLPs) to induce potent neutralizing Abs (Teoh et al., 2012; Fibriansah et al., 2014, 2015a,b). Notably, mAb 1F4 recognizes residues in DI/II hinge of an E monomer but cannot bind recombinant E protein, which primarily forms monomer at the concentration tested in the binding assay (Fibriansah et al., 2014). The difference in the angle of DI/II between virions and solution is likely to explain such discrepancy. In light of the observation that anti-prM mAbs are weakly or non-neutralizing and cause ADE (Huang et al., 2006; Dejnirattisai et al., 2010; Rodenhuis-Zybert et al., 2010, 2011), how to present E protein in the context similar to that on virions without pr or prM protein in the vaccine preparation will be challenging but promising as the next generation vaccine. This might require the approach of genetic engineering or epitope-modification to better present the immunogen and induce more potent neutralizing Abs and less enhancing Abs, thus reducing the risk of ADE. An example of epitope-modified vaccine was demonstrated by the fusion loop-mutated dengue DNA vaccine, which induced comparable neutralizing Abs but less enhancing Abs in mice compared with WT DNA vaccine (Crill et al., 2012; Hughes et al., 2012). Similarly, a recent study of fusion loop-modified ZIKV mRNA vaccine showed less enhancing Abs were induced (Richner et al., 2017).

The recent discovery of novel E-dimer epitopes recognized by human cross-reactive potent neutralizing mAbs raises the possibility that the task of inducing balanced neutralizing Abs against 4 DENV serotypes by tetravalent vaccines can be achieved by using E dimers to induce cross-reactive anti-E dimer epitope neutralizing Abs (Dejnirattisai et al., 2015; Rouvinski et al., 2015). However, the design of recombinant E dimers to present the right conformation of E-dimer epitope remains to be explored.

The observations that anti-fusion loop mAbs derived from secondary DENV infection have higher binding avidity and neutralizing potency compared with those derived from primary infection (Tsai et al., 2013) suggest that during secondary infection memory B cells recognizing the common epitopes expand rapidly to generate cross-reactive, high avidity, and potent neutralizing Abs through affinity maturation. This was supported by higher level and rapid increase in serum avidity, cross-reactive memory B cells and plasmablasts after secondary DENV infection compared with primary infection (Mathew et al., 2011; Wrammert et al., 2012; Xu et al., 2012; Zompi et al., 2012, references in Tsai et al., 2013). The recent report that cross-reactive anti-E Abs contributed significantly to neutralizing activities against both exposed and non-exposed DENV serotypes after secondary DENV infection not only provides an explanation for the multitypic neutralizing Abs (Tsai et al., 2015), but also suggests a strategy of sequential heterotypic immunization with two or three serotypes of live-attenuated dengue vaccine to mimic natural DENV infection and induce immunity against all four serotypes. The epidemiological observations of higher risk of DHF/DSS during secondary infection raise concerns on the possible ADE and severe disease after the second dose of sequential immunization. Notably, heterotypic immunization with monovalent live-attenuated DENV vaccine in 30 individuals revealed minimal increased viremia compared with primary immunization and no severe disease (Durbin et al., 2011). In addition, the recent findings that CYD-TDV vaccine on DENV-experienced individuals showed no severe diseases and higher vaccine efficacy compared with naive individuals (74.3−83.7 vs. 35.5−43.2%) oppose the safety concern of sequential heterotypic immunization (Sabchareon et al., 2012; Capeding et al., 2014; Hadinegoro et al., 2015; Villar et al., 2015).

## Conclusion

The moderate efficacy, low efficacy among dengue naïve, and increased risk of hospitalization among young children during the Phase 2b and 3 trials of the first dengue vaccine (dengvaxia from Sanofi Pasteur) highlight the need for a better understanding of immunity, in particular humoral responses, after natural DENV infection. We have reviewed more than 300 human mAbs against DENV E protein and potent neutralizing epitopes reported thus far, together with human anti-prM mAbs and mouse mAbs. We have also reviewed several in-depth analyses of polyclonal human sera following DENV infection. With various sophisticated technologies of generating human mAbs, it is expected that more potent neutralizing epitopes as well as non-neutralizing or enhancing epitopes will be identified. This information together with detailed analysis of different categories of neutralizing and enhancing Abs in polyclonal sera will provide not only new insights into our understanding of dengue protection and pathogenesis, but also strategies for better immunogen design to induce more potent neutralizing Abs and less enhancing Abs for next-generation dengue vaccine development. Building upon our increasing knowledge of human mAbs against DENV, future research should continue in-depth analysis on polyclonal sera comparing those from natural infection and immunization to fine tune the correlates or surrogates of protection of Abs, and also on the memory B-cells in combination with next-generation sequencing to better understand the Abs repertoire after DENV infection and immunization.

## Author contributions

WT performed experiments, analyzed data, and wrote manuscript. HL analyzed data. f96445117@ntu.edu.tw. WW designed the study, analyzed data, and wrote manuscript.

### Conflict of interest statement

The authors declare that the research was conducted in the absence of any commercial or financial relationships that could be construed as a potential conflict of interest.
